# Study on syndrome differentiation and treatment in the management of chronic stable coronary artery disease to improve quality of life

**DOI:** 10.1097/MD.0000000000012097

**Published:** 2018-09-07

**Authors:** Ying-Fei Bi, Jing-Yuan Mao, Xian-Liang Wang, Ya-Zhu Hou, Zhi-Qiang Zhao, Yong-Bin Ge, Xiao-Han Yu

**Affiliations:** a First Teaching Hospital Affiliated to Tianjin University of TCM; b Tianjin University of TCM, Tianjin, China.

**Keywords:** chronic stable coronary artery disease, quality of life, Traditional Chinese medicine

## Abstract

**Background:**

Chronic stable coronary artery disease (SCAD) is a general term for all kinds of coronary artery disease (CAD), which includes patients with chronic stable angina, old myocardial infarctions, and also stable condition after revascularization (i.e., percutaneous coronary intervention, coronary artery bypass graft). According to 2012 AHA/ACC guidelines, the objective of the treatment for SCAD is to maintain or recover patients’ exercise tolerance, quality of life, and avoid complication like heart failure, so as to decrease mortality, eliminate symptoms, and avoid adverse reactions. Traditional Chinese medicine (TCM) has 2000 years of history in managing CAD and has its advantages in improving quality of life. Using scientific method to evaluate, demonstrate, and conclude the clinical curative effect of TCM is an extremely important task for both TCM and integrative Chinese and Western medicine in the treatment of CAD.

**Methods:**

This research is to collect real effective cases from authoritative TCM cardiologists’ clinic, so as to build a TCM diagnosis and treatment information database that involve 2000 patients from 32 different top-3 hospitals of china. The primary outcome includes EuroQol-5 Dimensions and Four diagnostic method of TCM, and secondary outcome includes angina score and some laboratory indexes like electrocardiograms, dynamic electrocardiogram, ultrasonic cardiogram, and treadmill exercise testing. This research uses SPSS17.0. to do the statistical analysis application. Enumeration data use *χ*^2^ test and measurement data which fit normality test use *t* test. The analysis of drugs usage in different diseases, different syndromes and different life quality effect will use principal component analysis, factorial analysis, clustering analysis. and point mutual information method, and so on.

**Results:**

This research, based on past syndromes research and real clinical effective chronic SCAD cases, aim to build a TCM diagnosis and treatment information database.

## 1 Introduction

Chronic stable coronary artery disease (SCAD) is a general term for all kinds of coronary artery disease (CAD), which includes patients with chronic stable angina, old myocardial infarctions, and also stable condition after revascularization (i.e., percutaneous coronary intervention [PCI], coronary artery bypass graft [CABG]). According to 2012 AHA/ACC guidelines, the objective of the treatment for SCAD is to maintain or recover patients’ exercise tolerance, quality of life, and avoid complication like heart failure, so as to decrease mortality, eliminate symptoms and avoid adverse reactions.

Traditional Chinese medicine (TCM) has 2000 years of history in managing CAD and has its advantages in improving quality of life. Using scientific method to evaluate, demonstrate, and conclude the clinical curative effect of TCM is an extremely important task for both TCM and integrative Chinese and Western medicine in the treatment of CAD. Recent researches mainly focus on certain types or some certain physiopathology stages of CAD while they’re not comprehensive. Worse will, different regions experts have their own diagnosis and treatment plans. There is a lack of unified syndrome differentiation and prescriptions plan for the chronic stable CAD and not enough attention has been paid to TCM despite it having many advantages in improving quality of life in these patients. Considering all the above, this research studied past syndromes’ research results and real clinical effective chronic SCAD cases. Cases were analyzed and explored, effective cases were filtered, syndrome differentiation of the prescriptions was refined, thus building a TCM diagnosis and treatment information database.

## 2 Methods

**Figure 1:**
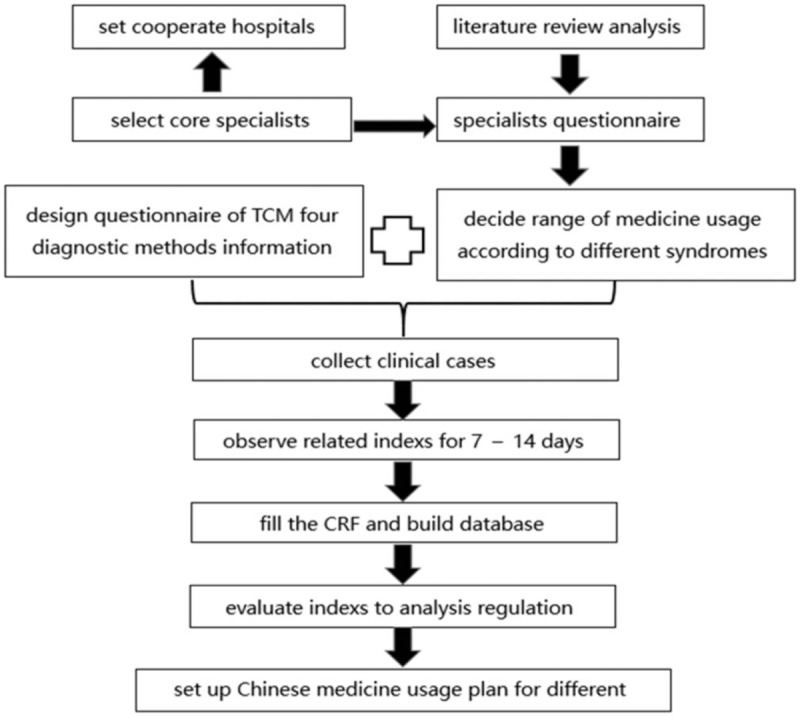
Flow chart of the search process.

The main purpose of this research is to build a TCM diagnosis and treatment database for chronic stable CAD, to summarize the regulation of TCM medicine usage and the effect of the medication in chronic stable CAD, to formulate a syndrome differentiation determined medicine plan based on the improvement of life quality in chronic stable CAD patients (Fig. 1). This research is registered in Chinese clinical trial registry on december 2, 2016, http://www.chictr.org.cn/showproj.aspx?proj=16534.

This research is to collect real effective cases from authoritative TCM cardiologists’ clinic, so as to build a TCM diagnosis and treatment information database that involves 2000 patients. The cases will be analyzed and summarized to conclude the pattern of TCM usage and the effect of use, thus formulating a TCM treatment plan based on syndrome differentiation to improve the quality of life in the chronic SCAD patients. To complete this project, following 4 steps are needed:

1. Standardized medicine usage plan for different syndromes. Based on the TCM syndromes’ research result of 8129 CAD patients from 40 integrated hospitals of China, common syndromes have been determined as: qi deficiency, blood stasis, phlegm retention, yin deficiency, qi stagnation, yang deficiency, accumulation of sthenia-heat and coagulated cold syndrome. Based on the literature review and experts’ questionnaires, frequently used medicine database has been build and the range of medicine usage has been defined. The standardized TCM treatment can be seen in the electrical CRF.
2. Collection of effective cases. Real-time electrical case system (electrical CRF) will record the diagnosis and treatment given by the TCM cardiologists. It will also record relevant information like patient's basic information, medical history, TCM four diagnostic methods information, quality of life, Western diagnosis, TCM diagnosis, therapies, laboratory examinations, TCM four diagnostic methods information changes after treatment, quality of life improvement, and so on.
3. Analysis of clinical data. A database has been built based on successful cases of TCM diagnosis and treatment of chronic SCAD, evaluating outcomes of quality of life and symptoms improvement after TCM treatment.
4. Formulate syndrome differentiation and treatment based clinical diagnosis and treatment. After analyzing the effective and invalid cases, the relationship of TCM characteristics between the medicine-disease, medicine-syndrome, and medicine-symptom was summarized. This is to further refine the characteristics of TCM syndrome differentiation to establish a SCAD treatment plan.

### 2.1 Research subjects

Research subjects are 2000 patients with chronic SCAD from 32 different top-3 hospitals of china.

### 2.2 Diagnostic criteria

#### 2.2.1 Western medicine diagnostic criteria

Western medicine diagnostic criteria was fixed with reference to 2007 Guideline for diagnosis and treatment of patients with chronic stable angina,^[1]^ 2010 Chinese specialists consensus on management on chronic SCAD,^[2]^ 2013 ESC guidelines on the management of SCAD,^[3]^ 2014 ACC/AHA/AATS/PCNA/SCAI/STS focused update of the Guideline for the Diagnosis and Management of Patients with Stable Ischemic Heart Disease.^[4]^

#### 2.2.2 TCM diagnostic criteria

TCM diagnostic criteria was fixed with reference to 1990 *TCM syndromes differentiation treatment criteria on coronary artery disease* set by Cardiovascular Society Branch of Chinese and Western Combination Union,^[5]^*1997 national standard of the People's Republic of China Clinic terminology of traditional Chinese medical diagnosis and treatment—Syndromes,*^[6]^*principles of guideline on new Chinese medicine clinical research*,^[7]^*diagnostics of Chinese medicine*,^[8]^*TCM internal medicine*,^[9]^ and the experts questionnaire about special TCM syndromes of CAD around China.

### 2.3 Inclusion criteria

1. Diagnose as chronic SCAD at first visit.
2. Heart function classification between II∼III (CCS).
3. Be willing to accept Chinese herbal decoction.
4. Sign an informed consent.

### 2.4 Exclusion criteria

1. Suffer from acute myocardial infarction, unstable angina or prior revascularization (i.e., PCI, CABG) in the latest 60 days.
2. Have such diseases as follows: refractory hypertension; severe arrhythmia (atrial fibrillation with rapid ventricular response, atrial flutter, paroxysmal ventricular tachycardia, among others); pulmonary heart disease, rheumatic heart disease, myocarditis, cardiomyopathy, aortic dissection, and pulmonary embolism.
3. Hyperthyreosis, cervical spondylosis, gallbladder-cardiac syndrome, gastroesophageal reflux, esophageal hiatal hernia, neurosis, climacteric syndrome, and other diseases which would cause chest pain.
4. Alanine transarninase or serum creatinine more than twice the normal reference value.
5. Hemoglobin <100 g/L or have severe hematological system disease.
6. Malignant tumor.
7. Pregnant, lactating women, or other women have reproductive requirement.
8. Psychosis or cognition impairment person.
9. Taken Chinese medicine decoction in recent 2 weeks.
10. Not take part in any other clinical trial.

### 2.5 Elimination criteria

1. Patients been found did not meet the trial requirement after enrolled.
2. Patients do not take the medicine as required.
3. Patients not following up the trial.
4. Patients off the trial without leaving anything useful.

### 2.6 Drop out criteria

1. Patients off the trial on their own will.
2. Main researcher does not think that the patients are suitable for the trial any longer.

### 2.7 Suspension criteria

1. Serious safety problem happens in the trial.
2. During the process of the trial, some big problems have been found about the design of this trial, or some deviations happened because of which the effect of the medicine cannot be evaluated.
3. Chief department of the trial decides to undo the trial.

### 2.8 Treatment plan

#### 2.8.1 Western routine treatment

Take the same western routine treatment during the research. The treatment is given with reference to *2007 China Guideline on Chronic Stable Angina Diagnosis and Treatment*^[1]^ and *2010 Chinese Specialists Consensus on Management of Chronic Stable Coronary Artery Disease*.^[2]^

#### 2.8.2 Chinese medicine treatment base on syndrome differentiation

##### 2.8.2.1 Range of the medicine use

Based on the result of former literature review and specialists questionnaire, research had defined the range of medicine which can treat chronic stable CAD.^[10]^

##### 2.8.2.2 Precautions to medicine use

1. Researchers can add some experimental medicine appropriately.
2. Component of Chinese medicine decotions limited in 16 kinds of herbs.
3. Avoid using other types of Chinese medicine product like Chinese patent medicine, Chinese injection medicine, and so on, in the same time.
4. Chinese medicine decoction should use some frequently used medicine instead of uncommon, endangered medicine.

##### 2.8.2.3 Methods of taking medicine and treatment course

One packet of warm decoction every morning and evening is taken, wherein treatment course is 7 to 14 days.

### 2.9 Outcome

#### 2.9.1 Primary outcome

##### 2.9.1.1 EuroQol-5 Dimensions

EuroQol-5 dimensions (EQ-5D) has been accepted as a simple, operable, convenient, and comprehensive tool which includes 2 sections (five-dimension [5D] healthy description system and EQ-VAS). The 5D covers mobility, self-care, usual activities, pain/discomfort, and anxiety/depression. Every dimension has 5 degrades: easy, a little, medium, difficult, and challenging. EQ-VAS is a 20-cm visual vertical scale, on the top of the scale is 100 point, and it represents the best condition in patient's mind; the bottom is 0 point, which represents the worst condition in patient's mind.

##### 2.9.1.2 Four diagnostic method of TCM

According to the TCM syndromes’ research result from 8129 CAD patients, 47 items of the common four diagnosis method in CAD has been confirmed. Inquiry part includes chest pain, radiating pain, palpitations, shortness of breath, panting, feeling weak, spontaneous sweating, night sweat, dizzy, tinnitus, insomnia, dream-disturbed sleep, restlessness, aversion to cold and cold limbs, a bitter taste in mouth, cough, heaviness of the body, dry eyes, blurred vision, thirst with desire for water drink, soreness and weakness in the lower back and knee joints, frequent sighing, a distending or wandering pain in hypochondriac area, constipation, inspection part include laziness in speaking and mental fatigue, dark and lusterless complexion, a pale-white or sallow complexion, overweight, abdominal obesity, and bluish-purple lip and mouth. The tongue diagnosis includes pale red with luster tongue, enlarged tongue, teeth-marked tongue, purple dark tongue, pale dark tongue, petechiae tongue, sublingual varicose veins, white coating, yellow coating, thin coating, thick coating, and greasy coating. Pulse taking and palpation part include slippery pulse, wiry pulse, thready pulse, and deep pulse. Inquiry, inspection and tongue diagnosis part can use 6-level evaluation criterion (no or disappear, light, mild, medium, severe, very heavy). Healthy tongue and pulse like pale red tongue, thin coating, white coating use yes or no to describe.

#### 2.9.2 Secondary outcome

##### 2.9.2.1 Angina score

Fill the score in accordance with the frequency, lasting time, pain degree, and the dosage of nitroglycerin.

##### 2.9.2.2 Laboratory indexes

1. Electrocardiograms (ECGs): Record heart rate, heart rhythm, PR interval, QRS interval, QT/QTc, ST segment descending rage, the summation of the ST segment descending rage, T wave inversion, and so on.
2. Dynamic electrocardiogram: Record average heart rate, minimum heart rate(minHr), maximum heart rate(maxHr), supraventricular premature beat (SPB), supraventricular tachycardia, SPB bigeminy, SPB trigeminy, ventricular premature beat (VPB), VPB bigeminy, VPB trigeminy, sinus arrest, number or long pause >2000 ms, and so on.
3. Ultrasonic cardiogram: Ultrasonic cardiogram includes some structure index about cardiac like left ventricular end diastolic diameter , left ventricular end systolic diameter, left atrial diameter, left ventricular diameter, right ventricular, left ventricular posterior wall depth, and interventricular septum; systolic index like left ventricular stroke volume, cardiac output, cardiac index, ejection fraction, and fractional shortening; diastolic index like the ratio of early diastolic E and late diastolic A.
4. Treadmill exercise testing: Record heart rate and blood pressure before and after exercise, level of exercise, metabolic equivalent, exercise lasting time, ST segment descending rage, and frequency of angina and maximum of the ST segment descending rage during exercise.

#### 2.9.3 Safety measurement

1. Blood routine test, urine routine test, hepatorenal function test and electrolyte test.
2. Evaluate adverse event.

### 2.10 Evaluate points

Before and after the treatment.

## 3 Implementation management and quality control

### 3.1 Researchers’ training

To make sure the research can goes well, researchers will get some training about content and implementing measure of research through conference, DVD or video and field guidance.

### 3.2 Set-up operating standard

Set up the operating standard of research to make sure the research can proceed successfully.

### 3.3 Supervise implementation process

#### 3.3.1 Self-check

Each research hospital assigns a quality controller to manage and supervise the dynamic research, which includes updating research progress, managing informed consent form, and filling the CRF. If any problem happened during the implementation, problem can be raised through wechat, telephone, E-mail, or fax to clinical research associate to guarantee the factuality and accuracy of the research result.

#### 3.3.2 Monitoring

Undertaking hospital is to set up a quality supervision and management group to conduct scheduled or no-scheduled monitor to supervise hospital either on the spot or on web, and write inspection report of the process to ensure that the factuality and standardization.

### 3.4 Compliance management

This part involves researchers’ and patients’ compliance. For researchers, they should fully understand the aim of the research, and be familiar with the implementation. For patients, after they agree to informed consent and cooperate with the research at the same time they will be provided with health education.

### 3.5 Information management

Each specialist's cases will record sequentially. For example: No. 01 expert records his first patient as 01–001; researcher should make sure the data type-in CRF accurately, immediately, and completely; keep the primary information and record; present the CRF after saving the checked data. The record cannot be modified after submission.

### 3.6 Database management

#### 3.6.1 Questioning database

After CRF has been submitted, inspector will check the CRF and then form a doubt case form (DCF) to list all problems. Researchers will get the send back DCF timely, for each question, they will find a proper explanation, or they’ll get DCF second time.

#### 3.6.2 Management database

CRF can be logged in on the spot and a self-check can be done later. Database is updated after submitting checked data. Undertaking research group will assign specific database management people to set up a unique management group, who will in charge of unified management.

#### 3.6.3 Analysis database

##### 3.6.3.1 Definite the analysis people

Intentional analysis: intention to treat collection is the gather of qualified case and unqualified case (except eliminated case). Intention analysis on incomplete cases is to do last observation carried forward (LOCF).

Per-protocol analysis: Statistical analysis will done on qualified cases, which complete the research.

Safety data analysis: safety data require at least 1 treatment, and then analyzing the adverse event rate.

##### 3.6.3.2 Statistic method

Use SPSS17.0. to do the statistical analysis application. Enumeration data use *χ*^2^ test and measurement data which fit normality test use *t* test. The analysis of drugs usage in different diseases, different syndromes and different life quality effect will use principal component analysis, factorial analysis, clustering analysis, and point mutual information method, and so on.

## 4 Discussion

TCM has 2000 years of history in managing CAD and has its advantages in improving quality of life. Using scientific method to evaluate, demonstrate and conclude the clinical curative effect of TCM is an extremely important task for both TCM and integrative chinese and western medicine in the treatment of CAD. Recent researches mainly focus on certain types or some certain physiopathology stages of CAD while they’re not comprehensive. Worse will, different regions experts have their own diagnosis and treatment plan. There is a lack of unified syndrome differentiation and prescriptions plan for the chronic stable CAD and not enough attention has been paid to TCM despite it having many advantages in improving quality of life in these patients. Considering all the above, this research studied past syndromes research results and real clinical effective chronic SCAD cases. Cases were analyzed and explored, effective cases were filtered, syndrome differentiation of the prescriptions was refined, thus building a TCM diagnosis and treatment information database.

## 5 Others

### 5.1 Ethic issue

This plan meet the requirement on trial subjects’ benefits and rights item in *Declaration of Helsinki*. This plan can be applied only after signed approval opinion by First Teaching Hospital of Tianjin TCM university ethic committee. If any change happened in this research like main content, informed consent and main researchers change during the process, ethic examine should be resubmitted by the ethic committee.

### 5.2 Trial status

The study has been recruiting patients since December 2016.

### 5.3 Declarations section

#### 5.3.1 Ethical Approval and consent to participate

This trial protocol was approved by the Ethics Committee of First Teaching Hospital of Tianjin University of TCM on November 16, 2016 (TYLL2016[K] 024). All subjects provided signed informed consent, and the study followed the Helsinki Declaration.

### 5.4 Consent for publication

The corresponding author has the right to grant on behalf of all the authors and does grant on behalf of all authors.

### 5.5 Availability of supporting data

All data were managed by the independent investigator of Tianjin University of TCM.

All study outcomes were evaluated by clinical operators. Statistical analysis was completed by statistics center of First Teaching Hospital of Tianjin University of TCM.

## Acknowledgements

We gratefully acknowledge the contributions of the following hospitals: Third Affiliated Hospital of Beijing University of Chinese Medicine, Xiyuan Hospital of China Academy of Chinese Medical Sciences, Guang’anmen Hospital of China Academy of Chinese Medical Sciences, Dongfang Hospital of Beijing University of Chinese Medicine, Dongzhimen Hospital Affiliated to Beijing University of Chinese Medicine, Beijing Hospital of Traditional Chinese Medicine, First Teaching Hospital of Tianjin University of TCM, Second Affiliated Hospital of Tianjin University of TCM, Shijiazhuang Traditional Chinese Medicine Hospital, Tianjin Academy of Traditional Chinese Medicine Affiliated Hospital, Shanxi Hospital of Traditional Chinese Medicine, Heilongjiang Provincial Hospital of traditional Chinese Medicine, Affiliated Hospital of Changchun University of Traditional Chinese Medicine, Affiliated Hospital of Liaoning University of Traditional Chinese Medicine, The Second Affiliated Hospital of Liaoning University of traditional Chinese Medicine, Shandong Province Hospital of Traditional Chinese Medicine, Jiangsu Province Hospital of TCM, Shuguang Hospital Affiliated to Shanghai University of Traditional Chinese Medicine, The First Affiliated Hospital of Anhui University of Traditional Chinese Medicine, Jiangxi Provincial Hospital of Chinese Medicine, The First Affiliated Hospital of Henan University of TCM, Henan Province Hospital of TCM, The First Hospital of Hunan University of Chinese Medicine, The Second Affiliated Traditional and Western Medicine Hospital of Hunan University of Traditional Chinese Medicine, Affiliated Hospital of Hunan Academy of Chinese Medicine, First Affiliated Hospital of Guangzhou University of Traditional Chinese Medicine, Guangdong Provincial Hospital of Chinese Medicine, Guangdong Second Troditional Chinese Medicine Hospital, The Second Affiliated Hospital of Shanxi University of Traditional Chinese Medcine, Shanxi Province Hospital of Traditional Chinese Medicine, The Affiliated Hospital to Shanxi University of Traditional Chinese Medicine, Affiliated Hospital of Gansu University of Chinese Medicine, and Traditional Chinese Medicine Hospital of Xinjiang Uygur Autonomous Region.

## Author contributions

Y.F.B., X.L.W., and Y.Z.H. took part in the design of the study and did the literature review; Y.B.G., Z.Q.Z., and X.H.Y. were responsible for clinical practice; J.Y.M. made substantial contributions to the conception and design of the study and took charge of drafting the study protocol essentially, is the corresponding author for the article. All authors read and approved the final manuscript.

**Conceptualization:** Jingyuan Mao.

**Data curation:** Yazhu Hou, Xiaohan Yu.

**Formal analysis:** Xianliang Wang, Yongbin Ge.

**Funding acquisition:** Yingfei Bi.

**Investigation:** Yingfei Bi, Zhiqiang Zhao, Xiaohan Yu.

**Methodology:** Yingfei Bi.

**Project administration:** Jingyuan Mao.

**Resources:** Yingfei Bi, Xianliang Wang.

**Software:** Yingfei Bi, Yazhu Hou, Yongbin Ge.

**Supervision:** Yingfei Bi, Yazhu Hou, Xiaohan Yu.

**Validation:** Yazhu Hou.

**Visualization:** Yazhu Hou.

**Writing – original draft:** Yingfei Bi, Xianliang Wang, Yazhu Hou, Yongbin Ge.

**Writing – review & editing:** Yingfei Bi, Xianliang Wang, Yazhu Hou, Yongbin Ge.
