# Study on syndrome differentiation and treatment in the management of chronic stable coronary artery disease to improve quality of life: Erratum

**DOI:** 10.1097/MD.0000000000015401

**Published:** 2019-04-19

In the article, “Study on syndrome differentiation and treatment in the management of chronic stable coronary artery disease to improve quality of life”,^[1]^ which appeared in Volume 97, Issue 36 of *Medicine*, the authors would like to note an updated affiliation for affiliation a. Affiliation a should appear as “First Teaching Hospital of Tianjin University of Traditional Chinese Medicine, Tianjin, China.”
